# An *in vivo *analysis of the localisation and interactions of human p66 DNA polymerase δ subunit

**DOI:** 10.1186/1471-2199-6-17

**Published:** 2005-07-06

**Authors:** J Richard G Pohler, Marit Otterlei, Emma Warbrick

**Affiliations:** 1 Department of Surgery and Molecular Oncology, University of Dundee, Ninewells Hospital and Medical School, Dundee DD1 9SY, UK; 2 Department of Cancer Research and Molecular Medicine, The Faculty of Medicine, The Norwegian University of Science and Technology, N-7489 Trondheim, Norway

## Abstract

**Background:**

DNA polymerase δ is essential for eukaryotic DNA replication and also plays a role in DNA repair. The processivity of this polymerase complex is dependent upon its interaction with the sliding clamp PCNA and the polymerase-PCNA interaction is largely mediated through the p66 polymerase subunit. We have analysed the interactions of the human p66 DNA polymerase δ subunit with PCNA and with components of the DNA polymerase δ complex *in vivo*.

**Results:**

Using the two-hybrid system, we have mapped the interaction domains for binding to the p50 polymerase δ subunit and with PCNA to the N-terminus and the C-terminus of p66, respectively. Co-immunoprecipitation experiments confirm that these interaction domains are functional *in vivo*. Expression of EGFP-p66 shows that it is a nuclear protein which co-localises with PCNA throughout the cell cycle. p66 is localised to sites of DNA replication during S phase and to repair foci following DNA damage. We have identified a functional nuclear localisation sequence and shown that localisation to replication foci is not dependent upon active nuclear import. Sub-domains of p66 act as dominant negative suppressors of colony formation, suggesting that p66 forms an essential structural link between the p50 subunit and PCNA. Analysis of the C-terminal PCNA binding motif shows that deletion of the QVSITGFF core motif results in a reduced affinity for PCNA, while deletion of a further 20 amino acids completely abolishes the interaction. A reduced affinity for PCNA correlates with reduced targeting to replication foci. We have confirmed the p66-PCNA interaction *in vivo *using fluorescence resonance energy transfer (FRET) techniques.

**Conclusion:**

We have defined the regions of p66 required for its interaction with PCNA and the p50 polymerase subunit. We demonstrate a functional link between PCNA interaction and localisation to replication foci and show that there is a direct interaction between p66 and PCNA in living cells during DNA replication. The dominant negative effect upon growth resulting from expression of p66 sub-domains confirms that the p66-PCNA interaction is essential *in vivo*.

## Background

The polymerisation of deoxyribonucleotides into DNA is one of the most fundamental processes of life and is carried out by DNA polymerases. A wide variety of these enzymes exist, including the accurate polymerases α, β, δ, ε and γ and also the more recently discovered translesion polymerases, which can show a more flexible substrate specificity [1]. The replicative polymerases all exist as multi-protein assemblies which show complex patterns of interaction, not only between individual subunits, but also with other components of the repair and replication machinery [1]. Many of the components of the DNA replication apparatus also show interactions with elements of the networks which regulate cell cycle progression and checkpoint control.

Chromosomal replication in eukaryotic cells requires three distinct DNA polymerases: α, δ and ε. The pol α-associated primase subunits synthesise oligonucleotides which are elongated for a short length (approximately 35 nucleotides) by the large catalytic subunit of DNA polymerase α. These short RNA-DNA segments are then elongated by pol δ or ε through a series of reactions in which the pol α-primase complex is displaced by Replication Factor C (RFC)-PCNA. The precise roles played by pol δ and pol ε are still not completely clear. Studies using purified protein preparations in SV40 DNA replication *in vitro *show that pol α and pol δ are sufficient for the completion of DNA replication, suggesting that pol δ acts as the major DNA replicative polymerase [2]. However, we also know that pol ε is located at or near the replication fork and evidence for a direct role for pol ε in replication has emerged from Xenopus cell free systems [3,4]. Pol δ plays an essential role in both DNA replication and repair: biochemical and genetic studies have implicated pol δ in mismatch repair [5], nucleotide excision repair [6], base excision repair [7,8] and double strand break repair [9,10]. The action of pol δ as a processive enzyme requires its interaction with proliferating cell nuclear antigen (PCNA) which functions as a molecular sliding clamp and which is loaded onto DNA by the action of the RFC complex [2,11,12].

Recent studies in fission yeast (*S. pombe*) and mammalian cells have shown that the native form of DNA polymerase δ consists of four subunits acting as a heterotetramer [13-15]. The large catalytic subunit, p125, forms a tightly associated heterodimer with the p50 subunit and this dimeric form of the enzyme has been extensively studied. The function of the smallest subunit (p12) is still poorly understood. In *S. pombe*, deletion of the p12 homologue, Cdm1, does not affect cell proliferation or cause sensitivity to DNA damaging agents [16]. *In vitro *reconstitution experiments using purified human proteins have shown that p12 is not essential for PCNA-dependent DNA replication, but that it is required to give a polymerase activity comparable to that of the native pol δ complex isolated from cell extracts [17-19]. In *S. pombe*, over-expression of Cdm1 can rescue temperature sensitive alleles in each of the genes encoding the other DNA polymerase δ subunits [16]. This is consistent with a model in which this subunit stabilises the pol δ complex.

Studies in *S. pombe *identified Cdc27 as a subunit of DNA polymerase δ. The catalytic subunit, Pol3, interacts directly with the *S. pombe *homologue of the p50 subunit, Cdc1 [20]. Cdc1 in turn interacts with Cdc27, which is the homologue of p66 [21]. Cdc27 interacts directly with PCNA and this interaction is mediated by a conserved PCNA binding motif [21,22]. Reconstitution of the subunits of DNA pol δ from *S. pombe *has shown that although the three-subunit polymerase complex shows low basal processivity, this is markedly increased by the addition of Cdc27 in PCNA-dependent *in vitro *assays. Both the three-subunit complex and four subunit complex containing Cdc27 in which the PCNA-binding motif has been deleted both required PCNA for processive polymerisation, suggesting a second site within the complex for PCNA interaction, possibly within the large catalytic subunit Pol3 [23]. Genetic experiments in *S. pombe *have shown that the ability of Cdc27 to bind simultaneously to Cdc1 (the p50 homologue) and to PCNA is essential for its biological function [21]. The p66 subunit and its homologues all contain a consensus PCNA-binding domain at the C-terminus which is homologous to that found in p21(WAF1/Cip1), Fen1, DNA ligase I, the large subunit of RFC and many other proteins involved in DNA replication, repair and modification [22]. Based on gel filtration experiments, the *S. pombe *polymerase δ complex was previously thought to exist as a dimer with Cdc27 mediating the dimer interface [15]. It is now known that these results were due to the highly asymmetrical shape of Cdc27 and that pol δ exists as a monomer [23]. Similar results have been found in *S. cerevisiae *[24].

The homologue of Cdc27 in *S. cerevisiae *is Pol32, although a homologue of Cdm1/p12 has not been found. *In vitro *experiments show that the addition of Pol32 to the purified dimeric form of the polymerase results in an increased processivity rate and Pol32 interacts with PCNA [25]. Surprisingly, Pol32 is not essential; deletion of the *POL32 *gene results in a temperature sensitive phenotype and sensitivity to DNA damage [26]. *POL32 *deletion is lethal when combined with a temperature sensitive mutation in *POL3 *which encodes the pol δ catalytic subunit. This suggests that, in contrast to *S. pombe*, the interaction between pol δ and PCNA can be mediated by some other mechanism in *S. cerevisiae*. In native gel analysis using purified proteins, a well-defined complex between polymerase δ and PCNA was only observed when Pol32 was present containing an intact PCNA-binding domain. However, in *in vitro *DNA replication assays, loss of the p50-binding domain had a far stronger effect than loss of the PCNA-binding domain on PCNA-dependent polymerase processivity. This suggests that a region in addition to the PCNA-binding consensus in Pol32 contributes to the interaction of PCNA with the polymerase complex [27].

A direct interaction between PCNA and the p125 subunit has been demonstrated in mammalian cells [17,28,29], although this interaction does not seem to exist in either *S. pombe *or *S. cerevisiae *and some studies have failed to find it in human cells [26,30-32].

In mammalian cells, the Cdc27 homologue (p66) was identified as a polymerase δ subunit by affinity chromatography using either PCNA or anti-p125 antibodies to isolate the polymerase complex [17,33,34]. Interaction studies using purified proteins have shown that p66 binds directly to PCNA and p50. p66 also stabilises the association between p125 and p50 and increases the overall affinity of the polymerase δ complex for PCNA [17,35]. p66 was found to stimulate DNA synthesis by three- to four-fold in the presence, but not in the absence of PCNA. In contrast, basal DNA synthesis by the p125-p50 dimeric complex is not stimulated by PCNA [32,35,36]. However, these results are at variance with results showing that the processivity of the dimeric p125-p50 form of the polymerase could be stimulated by PCNA [37]. Using purified proteins from *S. cerevisiae*, DNA replication by a dimeric complex of Pol3-Pol31, which is equivalent to p125-p50 in human cells, was PCNA-dependent, though proceeded inefficiently and was characterised by frequent pausing. Addition of Pol32, which is equivalent to p66, resulted in significantly increased PCNA-dependent processivity [25].

Here we describe results from our examination of the interactions of human p66 pol δ subunit with PCNA and with components of the DNA polymerase δ complex *in vivo*. We have mapped interaction domains by expressing various regions of the protein and also analysed their subcellular localisation. We have measured fluorescence resonance energy transfer (FRET) to determine if p66 forms direct, rather than complex-mediated, interactions *in vivo*.

## Results

### Two-hybrid analysis of p66-protein interactions

**Figure 1:**
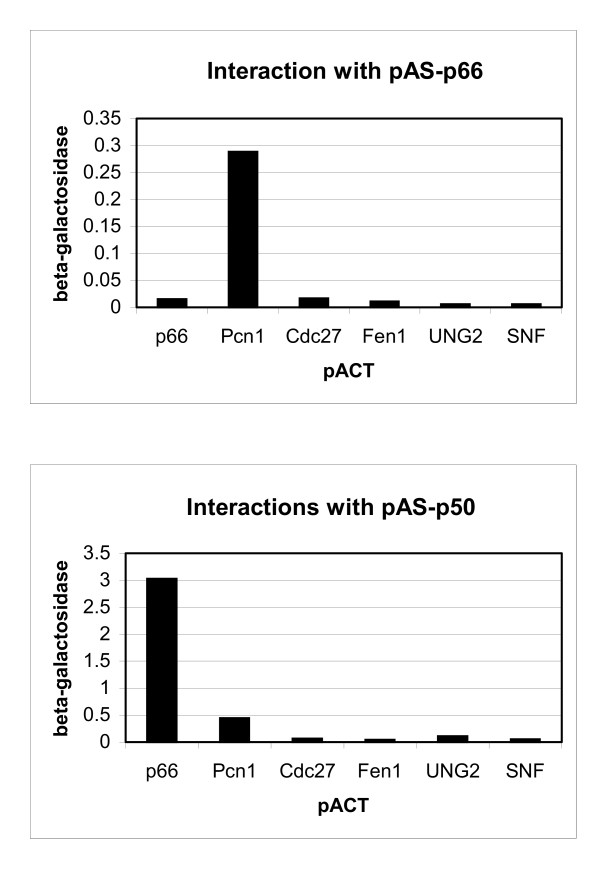
**Two-hybrid analysis of p66 and p50 interactions. **β-galactosidase assays were carried out as described in the Methods section using strain Y190 co-transformed with plasmids as indicated. Top panel: various proteins expressed as fusions with the transcription activation domain of GAL4 (pACT) were co-expressed with human p66 expressed as a fusion with the sequence specific DNA-binding domain of GAL4 (pAS). Lower panel: activation domain fusions were co-expressed with human p50 fused with the sequence specific DNA-binding domain of GAL4. pACT constructs are as described: p66 (Methods section); Pcn1 (*S. pombe *PCNA) [43]; Cdc27 (*S. pombe *p66 homologue) [21]; Fen1 (human repair and replication endonuclease) [45]; UNG2 (human uracil DNA glycosylase) [44] and *S. cerevisiae* Snf1 as a negative control.

Two-hybrid analysis was used to investigate the potential interactions of p66 with the p50 subunit and with PCNA. Constructs were made expressing full-length p66 as fusions either with the transcriptional activation domain of GAL4 (pACT-p66) or the DNA binding domain (pAS-p66). As shown in Figure 1, the fusion protein expressed from pAS-p66 showed a highly specific interaction with *S. pombe *PCNA. Human PCNA could not be used as a pACT fusion as this construct shows strong self-activation. There was no detectable p66-p66 interaction suggesting that the protein does not dimerise. This correlates with observations seen with *S. cerevisiae*: although the p66 homologue Pol32 was initially thought to dimerise in the two hybrid system it was later found that this was due to the bridging activity of the endogenous *S. cerevisiae *PCNA [24]. *S. pombe *Cdc27 cannot be analysed in this way as it results in reporter activation when expressed as a fusion with the GAL4 DNA binding domain (data not shown). No interaction was seen between p66 and Cdc27, or between p66 and any other proteins tested, including those known to interact with PCNA in the replication complex such as Fen1 or uracil DNA glycosylase (UNG2). Although expression of pAS-p50 results in some background reporter activation, it is clear that there is a significant interaction between the p50 and p66 subunits (Fig. 1, lower panel). This is also seen using pAS-p66 and pACT-p50 constructs (data not shown). No significant levels of interaction was seen between the *S. pombe *homologue of p66, Cdc27, and human p50. It seems likely that this reflects the low levels of homology between p66 and Cdc27, which is less than 24% overall [33].

To identify other proteins which interact with p66, a two-hybrid screen was undertaken with the full-length protein fused to the GAL4 DNA binding domain. 1.4 × 10^6 ^clones were screened in two separate experiments and two independent positive clones identified with strong activation of the reporters His3 and β-galactosidase. These clones showed a very specific interaction with p66 when compared with a range of pACT constructs and were both found to encoded full-length clones of p50. To map the domain of p66 responsible for the interaction with p50, various regions of p66 were tested in the two-hybrid system. The smallest clone which showed an interaction was that encoding amino acids 1 – 144 (pACT-p66S2) which is a region similar to, though slightly smaller than, the Cdc1-interacting region in Cdc27 in *S. pombe *[21].

### Interactions in human cells

**Figure 2:**
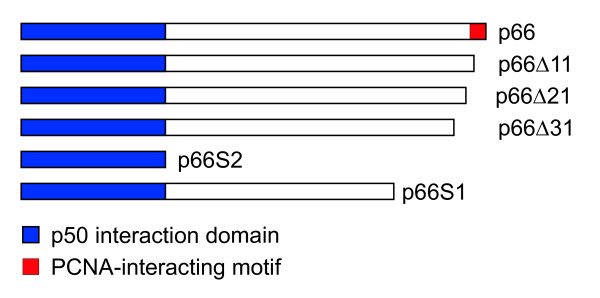
**Subclones of p66 expressed as Gal4 and EGFP fusions used for interaction analysis. **The regions of p66 shown were expressed in mammalian and two hybrid vectors and were made as described in the Methods section. The constructs p66Δ11, p66Δ21 and p66Δ31 have the C-terminal 11, 21 and 31 amino acids deleted, respectively. p66S2 expresses amino acids 1–144; p66S1 expresses amino acids 1–375.

**Figure 3:**
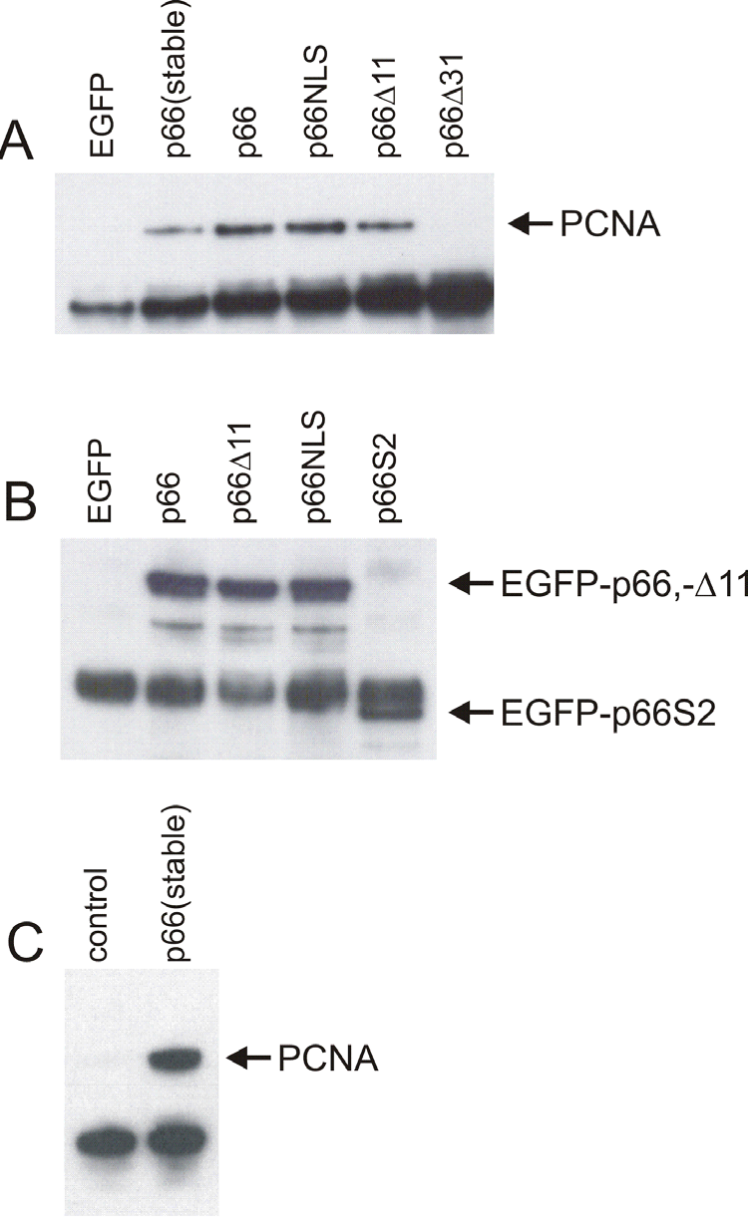
**Western blot analysis of co-immunoprecipitation experiments. **Extracts were prepared from cells expressing various EGFP-p66 fusion proteins as described in the Methods section and immunoprecipitation carried out as described. Cells were transfected with constructs expressing EGFP or EGFP fusions as indicated. "control" indicates non-transfected cells. Panel A: immunoprecipitation was carried with a rabbit polyclonal anti-EGFP antibody (Ab290, Abcam). Bound proteins were separated by SDS-PAGE and Western blot analysis carried out with a mouse anti-PCNA antibody (PC10). Panel B: immunoprecipitation was carried out with a rat anti-p50 antibody and the blot probed with a mouse anti-GFP antibody (Roche). Panel C: immunoprecipitation was carried out with a rat anti-p50 antibody and the blot probed with a mouse anti-PCNA antibody (PC10).

To study the interaction of p66 with PCNA further we used various constructs expressing p66 in human U2OS osteosarcoma cells as fusions with Enhanced Green Fluorescent Protein (EGFP). A conserved PCNA-binding domain is localised at the C-terminus of p66 and this is conserved between other p66 homologues [21,33-35]. Various C-terminal deletions of p66 were expressed as EGFP-fusion proteins (see Figure 2 for details of constructs) and a construct expressing a full length EGFP-p66 fusion protein mutated in a predicted nuclear localisation sequence also tested (p66NLS; see below for details). Immunoprecipitation was carried out from soluble cell extracts using an anti-EGFP antibody. Interaction with endogenous PCNA was analysed by Western blotting with an anti-PCNA antibody (Figure 3a). These results show that PCNA co-immunoprecipitates with full length p66 when fused to EGFP. We used both transiently transfected EGFP-p66 and a stable cell line expressing this construct. The slightly higher level of PCNA seen with the transiently transfected cells reflects the higher levels of EGFP-p66 expressed (data not shown). We were also able to show co-immunoprecipitation of myc-tagged p66 with PCNA using the polyclonal rabbit anti-PCNA polyclonal antibody 3009 (data not shown). Compared to transient transfection of the full-length construct, deletion of the C-terminal 11 amino acids which contains the conserved PCNA-binding domain QVISITGFF reduced, though did not completely abolish the interaction of the EGFP-tagged p66 with PCNA. However, deletion of the C-terminal 31 amino acids abolishes the interaction completely. The levels of expression of the full-length p66, Δ31 and Δ11 EGFP fusion constructs were indistinguishable in these experiments (data not shown). The N-terminal 144 amino acids which interacted with p50 in the two-hybrid system show no detectable interaction with PCNA (data not shown). Immunoprecipitation of p50 from these extracts followed by detection of EGFP by Western blot confirms the two hybrid data that the region composed of amino acids 1–144 expressed by pEGFP-p66S2 is sufficient for the interaction with p50 (Figure 3b). These results confirm that the N-terminal 144 amino acids of p66 is sufficient for the interaction with p50 and show that the interaction of p66 with PCNA is mediated *in vivo *by the conserved PCNA-binding motif, though N-terminal flanking sequences are also important. They show that p66 can interact with p50 independently of its interaction with PCNA. We also show that p66 mutated in a putative nuclear localisation sequence is capable of binding to both p50 and PCNA (see below).

Further evidence to support the role of p66 of mediating the p50-PCNA interaction is shown in Figure 3c. Anti-p50 antibodies were used for immunoprecipitation from either untransfected cells or those stably expressing EGFP-p66. It can be see that the amount of PCNA co-immunoprecipitated is significantly increased in the cell expressing the increased levels of p66. This suggests that p66 acts to mediate or otherwise stabilise the interaction between p50 and PCNA, and that the level of p66 in the cell is limiting for this process.

**Figure 4:**
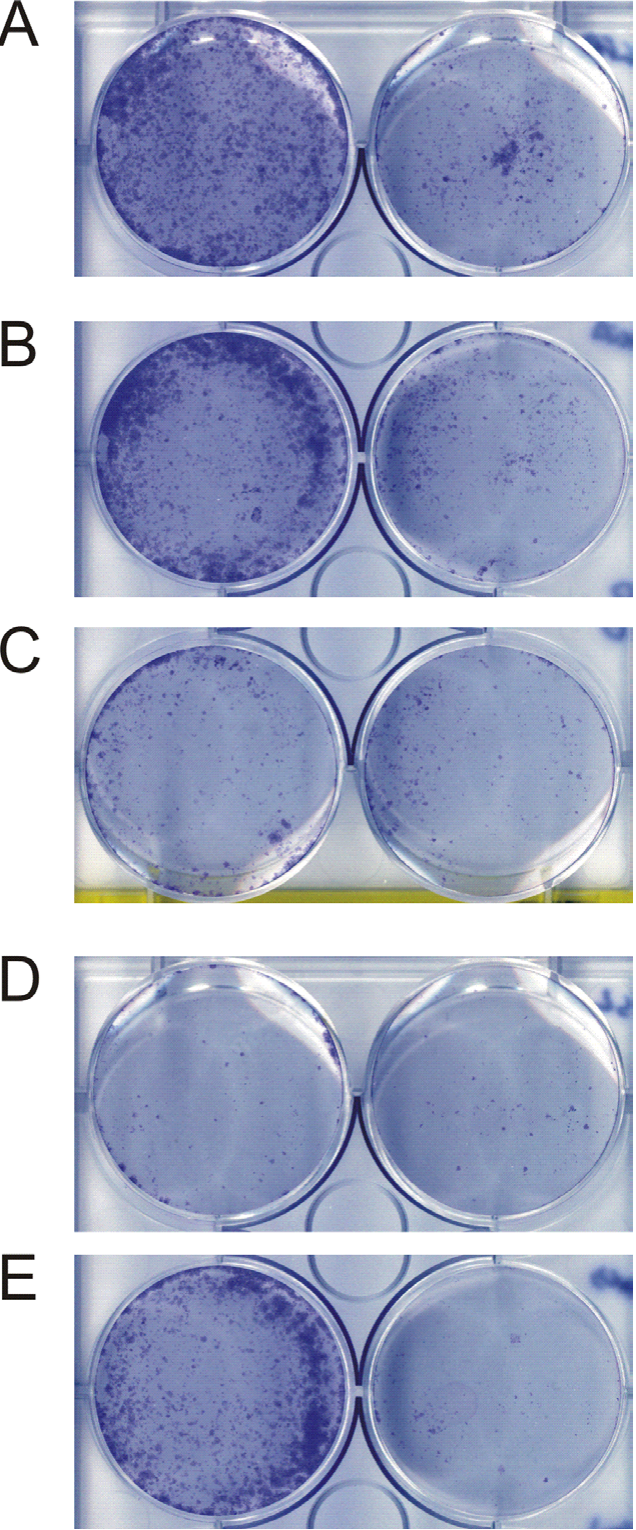
**Subdomains of p66 can act as dominant negative suppressors of proliferation in clonogenic assays. **Clonogenic assays were carried out to assess the effect of various p66 constructs on colony formation in U2OS cells; see the Methods section for details. Cells were plated at densities of 5 × 10^4 ^(left hand side) and 2.5 × 10^4 ^per well (right hand side) prior to transfection with EGFP constructs and grown under selective conditions for 10 to 20 days before being fixed and stained with Giemsa. The figure shows a representative result. Panel A: pEGFP; panel B: pEGFP-p66; panel C: pEGFP-p66Δ11; panel D: pEGFP-p66S2; panel E: pEGFP-p66NLS.

We have shown that the p66 protein interacts with p50 and PCNA and mapped the regions involved in these interactions. We predicted that high-level expression of regions of p66 which interact with only one of p50 or PCNA would be deleterious to the cell by acting as a dominant negative. To investigate this phenomenon we undertook clonogenic assays to determine if expression of various domains was deleterious to the cell over a period of time. Our results show that while expression of EGFP-p66 was indistinguishable from the EGFP control, expression of either EGFP-p66Δ11 or EGFP-p66S2 which do not interact with PCNA, results in reduced cell proliferation following selection for plasmid maintenance (Figure 4).

### Subcellular localisation of p66

**Figure 5:**
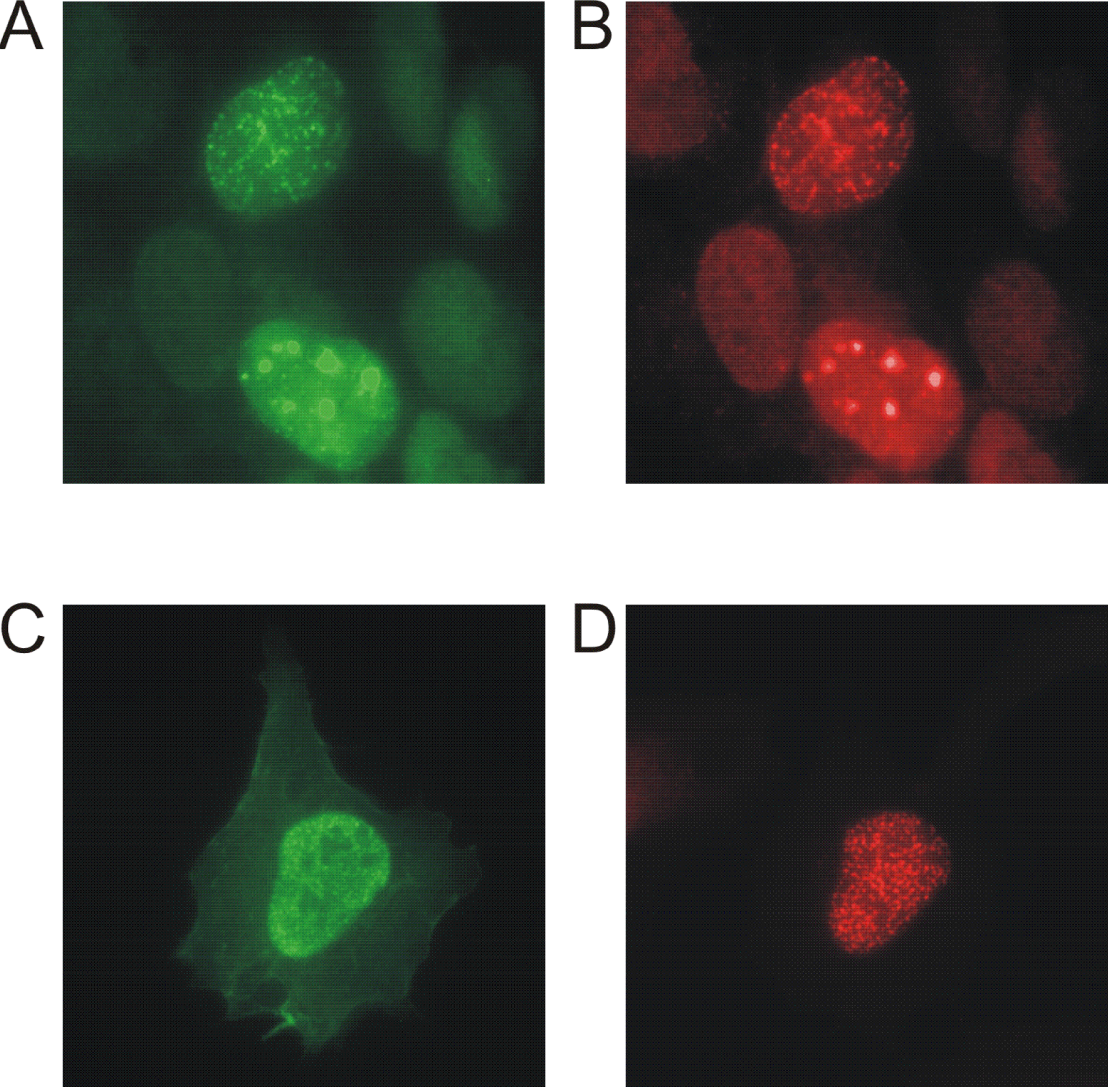
**p66 is directed to the nucleus by a single nuclear localisation sequence and is located to sites of DNA repair following UV damage. **U2OS cells were fixed and stained as described in Materials and Methods. Panels A and B: cells transiently transfected with the pEGFP-p66 construct were treated with 40 Jm^-2 ^UV at 254 nm and fixed 30 minutes post-treatment. Panel A: EGFP-p66 detected by an anti-EGFP antibody (Roche) (green); panel B: PCNA detected by staining with the anti PCNA-antibody 3009 (red). Panels C and D: cells transiently transfected with the pEGFP-p66NLS construct. Panel C: EGFP-p66NLS detected with the anti-EGFP antibody (green); panel D: cells stained with the anti-PCNA antibody as described above.

**Figure 6:**
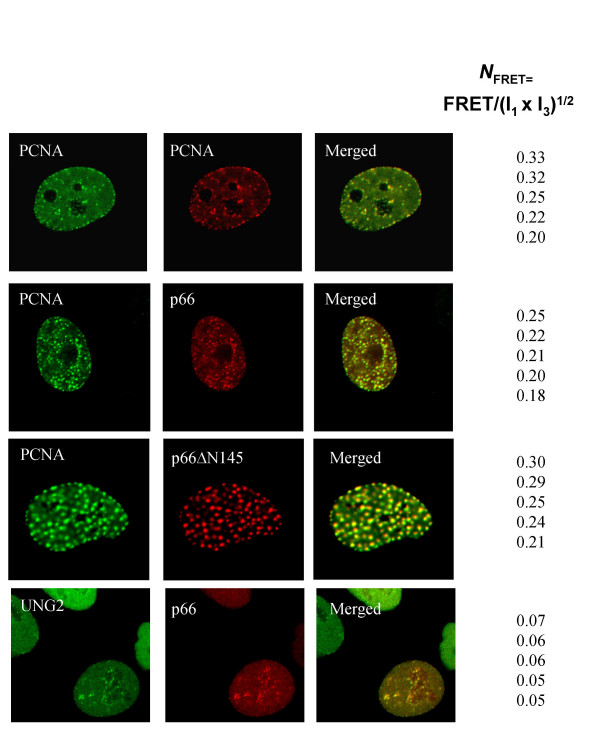
**FRET analysis of p66 and PCNA co-localisation **FRET analysis was carried out as described in the Methods section. Cells were co-transfected with two constructs as shown. PCNA was tagged with both ECFP and EYFP shown in green and red, respectively. p66 and p66Δ N145 were tagged with EYFP (shown in red) and UNG2 with ECFP (shown in green). Five of the representative high FRET values found within the given levels of intensities (donor intensities (I_1_, I_D1_) between 85–190, and acceptor intensities (I_3_, I_A3_) between 70–190. *N*_FRET _represents FRET normalized against protein expression levels. FRET is calculated from the mean of the intensities within one region of interest (ROI) containing more than 25 pixels (*i.e*., one replication focus). Within ROI, all individual pixels had intensities below 250. More than 90% of the UNG2-p66 co-localising foci did not FRET (FRET<0, see the Methods section).

During S phase, p66 localised to distinct nuclear spots which are typical of replication foci (see below; Figure 6). These foci or factories represent a conglomeration of proteins involved in DNA replication and post-replicative processing which are brought together at sites of DNA replication [38,39]. We also found that, following DNA damage outside S-phase, p66 was localised to large foci within the nucleus which are typical of sites of DNA repair. This is confirmed by the co-localisation of PCNA to these foci. Figure 5 (panels A and B) show cells fixed 30 minutes after a UV dose of 40 Jm^-2^. The upper cell shows a pattern of p66 and PCNA localisation which is typical of S phase, while the lower cell shows a pattern typical of DNA repair. This pattern of localisation is consistent with the role of DNA polymerase δ in repair, especially nucleotide excision repair, and confirms that the p66 subunit is specifically involved.

Analysis of the p66 protein sequence with the PredictNLS program lead to the identification of a single nuclear localisation sequence consisting of amino acids 319 – 326 (KKRRRIKL) [40]. Using site-directed mutagenesis, we created the plasmid pEGFP-p66NLS which expresses EGFP-p66 with the residues 320–322 (KRR) are mutated to NGG. This EGFP-p66NLS fusion protein was no longer exclusively localised to the nucleus, but was distributed throughout the nucleus and cytoplasm, indicating that this sequence represents a functional nuclear localisation sequence (Figure 5, panels C and D). Interestingly, this protein is still capable of localising to replication foci during S phase and could still interact with both PCNA and p50 (Figure 3), indicating that specific nuclear targeting is not a prerequisite for these processes. High levels of expression of this protein were not significantly deleterious to cell growth in colony assay experiments, suggesting that this NLS mutant was still fully functional and did not act as a dominant negative (Figure 4).

Many proteins have been identified which interact with PCNA through the small conserved PCNA-binding motif [22]. In some cases, this motif has been identified quite independently as a targeting sequence for localisation to replication foci [41] and a model has been proposed in which PCNA acts as an assembly platform for the replication machinery [39]. The conserved PCNA-binding motif in p66 is located at the extreme C-terminus, and we have already shown that this sequence is involved in the interaction of p66 with PCNA. To investigate the role of this domain in p66 sub-nuclear localisation, cells were transformed with the pEGFP-p66Δ11 and pEGFP-p66Δ31 constructs. These proteins were localised to the nucleus, but did not show a punctate pattern of localisation (data not shown), suggesting that they were not exclusively localised to replication foci. We have already shown that deletion of the last 10 amino acids significantly, though not completely, abolishes the interaction with PCNA while deletion of the last 30 amino acids completely abolishes this interaction. Our results indicate that complete abrogation of the p66-PCNA interaction is not required to abolish replication foci targeting. It also demonstrates that the interaction of p66 with PCNA is not required for nuclear localisation.

Since proteins that co-localise do not necessarily interact directly, we explored whether EYFP-p66 and ECFP-PCNA directly interact *in vivo*. To do this we used fluorescence resonance energy transfer (FRET) technology to determine whether the fluorescent tags (ECFP and EYFP) are closer than 100 Å/10 nm [42]. FRET occurs only when the intensity of emitted light measured in the presence of two fluorescently tagged proteins are greater than emitted light from cells transfected with ECFP- or EYFP-tagged proteins alone (i.e. background levels). The details of the calculations used to analyse the FRET signal are described in the Methods section. It is expected that, in at least some replication forks within a replication focus, ECFP and EYFP tagged PCNA will bind adjacently in the trimeric PCNA clamp, thus tagged PCNA were used as positive control. Furthermore, since no interaction between UNG2 and p66 could be detected in the yeast two-hybrid assay and there has been no report of an interaction between UNG2 and p66, we used ECFP-UNG2 and EYFP-p66 as a negative control for the analysis of these *in vivo *interactions in human cells. We calculated FRET from the mean intensities within a replication focus (the region of interest, ROI) and normalized these values against the different protein expression levels, N_FRET_, in cells co-transfected with ECFP-PCNA and EYFP- p66, ECFP-PCNA and EYFP-PCNA (positive control) and ECFP-UNG2 and EYFP-p66 (negative control) (Figure 6). The N_FRET _levels of five representative replication foci in the higher range of FRET values are given. Varying N_FRET _levels among different replication foci are as expected found since replication foci are dynamic structures and in addition endogenous proteins will compete with the tagged proteins for interactions. We find that the N_FRET _levels between PCNA and p66 are similar to N_FRET _between PCNA-PCNA. p66, PCNA and UNG2 are all localised to replication foci. In contrast, the N_FRET _levels observed between PCNA and p66 are four to five fold higher than between UNG2 and p66, indicating that PCNA and p66 are not merely co-localised to the same complex, but show a direct physical interaction in living cells. To confirm that this interaction is not merely due to the presence of both p66 and PCNA at the replication fork, we show that there is still a positive FRET interaction between PCNA and a p66 mutant which lacks the p50 interaction domain (p66DN145). By using FRET we can clearly distinguish between co-localisation and interaction.

## Discussion

Several lines of conflicting evidence exist concerning the mechanism of interaction of DNA polymerase δ with PCNA. The catalytic subunit p125 shows low activity and little response to PCNA stimulation when over-expressed in insect cells. Although direct interaction between PCNA and the p125 subunit appears to take place in mammalian cells [17,28,29] this either does not occur or is extremely weak in yeasts [26,31,32]. More recent work has shown that the major interaction between PCNA and the DNA polymerase complex is mediated by the third subunit, which is p66, Cdc27 and Pol32 in mammalian cells, *S. pombe *and *S. cerevisiae*, respectively. p66 homologues have now been identified in a wide range of organisms, but in general they have a very poor level of protein sequence similarity with the exception of the conserved PCNA-binding motif at the C-terminus.

Here we describe the analysis the protein interaction domains of human p66 by expressing various tagged and fusion proteins in cultured cells and we have correlated their subcellular localisations with their interactions *in vivo*. In our initial experiments using the two-hybrid system we were able to show that p66 was able to interact with both p50 and PCNA. This is similar to results seen in the *S. pombe *and *S. cerevisiae *homologues of p66. We also show that p66 does not dimerise in this system. In a two-hybrid screen using p66 as bait, the only positive clones identified were those expressing full-length p50. We did not expect to identify human PCNA in this screen, as the full-length protein when fused to the Gal4 activation domain results in a construct that is self-activating and so would be excluded as a false positive. The p50-interacting domain was mapped to amino acids 1–144, which is similar, though slightly smaller than the Cdc1 interaction region in Cdc27.

Using various domains of p66 expressed as fusions with EGFP in human cells, we were able to confirm that the domain identified as interacting with p50 using the two-hybrid system was also sufficient for the interaction in cell extracts using co-immunoprecipitation experiments. This domain showed no detectable interaction with PCNA. We also found that high levels of expression of either the p50-interacting or the PCNA-interacting domains are deleterious to cell proliferation over periods of growth selection in colony assays. This indicates that these domains can act as "dominant negatives" to compete for binding to the endogenous p50 and PCNA.

Fine mapping of the PCNA-binding domain at the C-terminus showed that the p66Δ11 protein (which has the minimal conserved PCNA-binding domain QVSITGFF plus QRK deleted) significantly reduced, though did not completely abolish binding to PCNA in co-immunoprecipitation assays. The deletion of an additional 20 amino acids completely abolished the interaction, suggesting a region N-terminal to the core-conserved domain makes a significant contribution to the PCNA interaction. The best-characterised incidence of a PCNA-interacting motif is in the cell cycle regulatory protein p21^WAF1^. The crystal structure of the PCNA-binding region from this protein complexed with PCNA has been solved which shows that the conserved motif makes a helical turn which has interactions within a hydrophobic pocket on PCNA. Sequences C-terminal to the motif make a β-sheet interaction with the inter-domain linker region of PCNA. In proteins such as DNA ligase I and the largest subunit of RFC the conserved motif lies at the extreme N-terminus indicating that N-terminal regions do not play a role in the PCNA interaction. However, in p66 we see that sequences lying up to 20 amino acids N-terminal to the conserved motif clearly play a role in the interaction. The mechanism by which this region interacts with PCNA awaits further investigation. The conserved PCNA-binding motif has been linked to replication foci targeting: in DNA ligase I the N terminal 20 amino acids containing the PCNA-binding motif are capable of directing localisation of heterologous proteins to replication foci [41]. However, we show here that the p66-Δ11 construct, which is still capable of binding to PCNA, does not appear to be targeted to replication foci. This result is difficult to interpret, as it may be that a small proportion of it, corresponding to the smaller amount seen binding to PCNA, is present in replication foci, but the amount is too low to be observed microscopically. It is clear that we see a correlation between PCNA binding and localisation to replication foci, with both these phenomena mediated by the interaction of p66 with PCNA through the conserved PCNA-binding motif.

These results correlate with those found for the p66 homologue, Cdc27, in *S. pombe *where binding to PCNA and protein function *in vivo *could be correlated. Reynolds et al. showed that the C-terminal 20 amino acids of Cdc27 were essential both for PCNA binding and protein function. However, high level expression of a construct lacking the C-terminal 10 amino acids containing the core PCNA-binding motif was still able to rescue growth of a *cdc27Δ *strain, though quite poorly. This suggests that regions of Cdc27 N-terminal to the core PCNA binding domain may also contribute to PCNA binding [21].

We have shown that p66 is a nuclear protein which is excluded from the nucleolus and is localised to replication foci during S phase. This pattern of localisation is shared with many proteins involved in DNA replication such as PCNA, pol α, Fen1, DNA ligase I, RFC, RPA etc. [38,39]. This pattern of co-localisation does not in itself show that the proteins make a direct physical interaction. To investigate this, we have used FRET analysis to substantiate a direct interaction between p66 and PCNA *in vivo*. We find that the N_FRET _between PCNA and p66 is similar to the N_FRET _shown by the extremely stable PCNA-PCNA interaction, (Figure 6) and are considerably higher than between proteins that co-localise, but do not interact, such as UNG2-p66. These results, together with the two hybrid and co-immunoprecipitation data, strongly support a direct physical interaction between p66 and PCNA *in vivo*.

In cell lines which stably express EGFP-p66 in addition to endogenous p66 we see an increased amount of PCNA associated with p50 compared to cells containing endogenous levels of p66. Since we already know that p66 can interact with both p50 and PCNA, this is strongly suggestive that the increased levels of p66 are stabilising the interaction. This implies that p66 mediates this interaction and that the level of p66 is a limiting factor for the amount of the polymerase complex associated with PCNA. This raises the interesting possibility that levels or localisation of p66 may play a regulatory role in DNA replication.

## Conclusion

Previously published work on the human p66 DNA polymerase δ subunit has shown that it forms part of the polymerase complex and interacts with PCNA in cell extracts [17,33,34]. p66 has been shown to bind p50 and PCNA using purified proteins [17,35]. In this work, we show that p66 interacts with p50 and PCNA using human cell extracts, and have mapped the protein domains involved in this interaction *in vivo*. The dominant negative effect upon proliferation shown by p66 constructs which cannot bind to PCNA suggests that the p66-PCNA interaction is an essential one. We also show that p66 is located to sites of DNA synthesis following DNA damage, which demonstrates that this subunit is present in the form of polymerase δ which is involved in DNA repair. We have identified a nuclear localisation sequence and show that p66 localisation to replication foci during S phase is not dependent upon active nuclear import. Finally we show that p66 is localised to replication foci in living cells using FRET techniques and show that it interacts directly with PCNA in these complexes.

## Methods

### Expression constructs

All DNA fragments made by PCR amplification were verified by DNA sequencing. The human p66 open reading frame was cloned from the human cDNA clone HA2030 supplied as by Takahiro Nagase at the Kazusa DNA Research Institute as a NcoI – BglII fragment to give the plasmids pAS-p66 and pACT-p66. The pAS-p50 clone (pGBT) was a kind gift from Dr. Stuart MacNeill, University of Edinburgh. The pACT-p50 clone was identified by two-hybrid screening. pACT-p66S2 (which expresses amino acids 1 – 144) was constructed by digesting pACT-p66 with SacI and religating. pACT-p66S1 (which expresses amino acids 1 – 375) was constructed by cloning the SacI fragment interior to the p66 open reading frame into pACT-p66S2 to extend the open reading frame. Clones of *S. pombe *PCNA in pAS and pACT are as previously [43]. Clones of UNG2 are as described [44]. Clones of Fen1 are as described [45].

A NcoI – XhoI fragment was cloned from HA2030 into pEG202 to give pEG-p66. A BamHI – XhoI fragment was cloned from pEG-p66 into pEGFP-C3 (Clonetech) digested with BglII – SalI to give pEGFP-p66 which expressed full-length p66 fused to EGFP. Similarly, the insert from pACT-p66S2 was cloned as an NcoI – XhoI fragment into pEG202 to give pEG-KIA-S2 and the insert from this plasmid cloned as an EcoRI – SalI fragment into pEGFP-C3 to give pEGFP-p66S2. The deletion construct pECFP-p66Δ11 which does not express the C-terminal 11 amino acids of p66 was amplified by PCR as a *Bgl*II-*Hin*dIII fragment and cloned into pECFP-C1. Further C-terminal truncations were generated from the original construct, using the QuikChange site-directed mutagenesis method (Stratagene) to incorporate stop codons. pEYFP-p66ΔN145 contains a HindIII – BamHI fragment created by PCR cloned into pEYFP-C1. The cloning of ECFP-PCNA, EYFP-PCNA and UNG2ECFP constructs is as previously described [46].

### Two hybrid screening

Two-hybrid analysis of proteins that interact with p66 was carried out essentially as previously described using the *S. cerevisiae *strain Y190 which expresses LacZ and His3 reporter constructs under the control of the Gal1 promoter [43]. For two-hybrid screening, Y190 cells containing pAS-p66 were further transformed with a human cDNA library in pACT, which was a gift from Steve Elledge. Putative interacting clones were identified by their ability to grow on media containing 50 mM 3-aminotriazole (3-AT), and tested for expression of the LacZ reporter by a filter lift assay for β-galactosidase activity. For β-galactosidase assays, 5 ml of culture log phase culture was washed once in water and the cell pellets permeablised by repeated immersion in liquid nitrogen. At least 3 independent transformants were analysed in each case. The pellets were resuspended in 1 ml of Z buffer containing 3% Brij 35 (Sigma) at 30°C and 0.2 ml of 4 mg/ml ONPG in Z buffer added. The reaction was stopped by the addition of 0.5 ml 1 M Na_2_CO_3_, cell debris was removed by centrifugation, and the OD_420 _of the supernatant measured. Units of β-galactosidase were calculated as: Units = (1000 × OD_420_)/(t × v × OD_600_) where t = time of reaction in minutes and v = volume of culture in ml.

### Cell culture and transfection

Cells were grown at 37°C and 5% carbon dioxide, in a humidified atmosphere. All cell types were grown in DMEM (PAA Laboratories, GmbH), supplemented with 10% v/v Foetal Calf Serum (FCS), 100 units/ml penicillin and 100 μg/ml streptomycin. Cells were transfected using Lipofectamine 2000 (Invitrogen) or using a calcium phosphate technique (Profection, Promega), according to the manufacturer's protocol, and harvested 24 hours post-transfection.

### Preparation of cell lysates and immunoprecipitation

Cells were transfected in 100 mm^2 ^dishes, and harvested by scraping into ice-cold PBS, followed by centrifugation at 1500 rpm for 3 minutes. Cell pellets were lysed using NP40 lysis buffer (50 mM TrisHCl (pH 8.0), 150 mM NaCl, 1% (v/v) NP-40) containing Complete™ protease inhibitors (Roche Biochemicals), and incubated at 4°C for 30 minutes, followed by centrifugation at 14000 rpm for 15 minutes. The resulting supernatants were decanted into a fresh tube and stored at -20°C until used. Protein concentrations were measured at 595 nm using the BioRad protein assay reagent (BioRad). The antibodies used for immunoprecipitation were rabbit polyclonal anti-PCNA (3009, Moravian Biotechnology Ltd.), rat monoclonal anti-p50 (7B4, kindly provided by Dr Heinz-Peter Nasheuer) and rabbit polyclonal anti-GFP (Ab290, Abcam). For each immunoprecipitation (IP), 1 μl of purified antibody, or 50 μl of tissue culture supernatant, was added to 750 μg of soluble protein, previously pre-cleared against protein G, and incubated overnight at 4°C with gentle agitation. 20 μl of protein G slurry (50% (v/v) in PBS) was added and incubated at 4°C for 1 hr with gentle agitation. The beads were pelleted by centrifugation at 2000 rpm for 3 minutes, and subsequently washed extensively with lysis buffer, prior to final resuspension with 20 μl of SDS loading buffer.

### SDS-PAGE and western blotting

Electrophoresis was performed using the NuPage system (Invitrogen). Either 10% or 4–12% Bis-Tris gradient gels were used and run with MES buffer as supplied by the manufacturer. Proteins were transferred to Immobilon-P membranes (Millipore) using the conditions directed by the manufacturer. Blots were blocked with 5% (w/v) milk in PBS for 1 hour at room temperature and then rinsed twice with PBS. Primary antibodies were diluted in 2% milk in PBS and added to the blot at a concentration of 2 μg/ml of purified antibody or 1:1000 ascites/serum. This was incubated for 1 hour at room temperature followed by extensive washing with PBS 0.2% Tween-20. HRP-conjugated secondary antibodies were added, incubated and washed as for primaries. Primary antibodies used were mouse monoclonal anti-PCNA antibody (PC10) and mouse monoclonal anti-EGFP antibody (Roche). Peroxidase-coupled donkey anti-mouse IgG (Jackson Immunoresearch) was used as secondary antibody. Immunoblots were visualised by enhanced chemiluminescence (ECL, Amersham Pharmacia), according to the manufacturers instructions.

### Immunofluorescence

Cells were grown and transfected in 2-well chamber slides (Lab Tek). Cells were washed twice with ice-cold PBS, and fixed with 4% (v/v) paraformaldehyde for 5 mins at room temperature (RT), followed by another PBS wash. Cells were subsequently permeabilised with 0.2% Triton X-100 in PBS for 10 minutes at RT. The primary antibodies used were rabbit polyclonal anti-PCNA (3009, Moravian Biotechnology Ltd.) diluted 1 in 5000, and mouse monoclonal anti-EGFP (Roche Biochemicals) diluted 1 in 500. Secondary antibodies used were FITC-coupled donkey anti-mouse, and Texas Red coupled donkey anti-rabbit (both Jackson Immunoresearch), diluted as recommended. All antibodies were diluted with 3% BSA in PBS.

Primary antibodies were added for 1 hour at 4°C. After washing twice with 3% BSA in PBS, secondary antibodies were added for 1 hour at 4°C, diluted as before. After washing twice with PBS, nuclei were stained with DAPI (Sigma) at a final concentration of 0.5 μg / ml in PBS. Cells were washed again in PBS before mounting with one drop of Hydromount (National Diagnostics) containing 2.5% (w/v) DABCO anti-fade (Sigma) and visualised on a Zeiss MC100 fluorescence microscope.

### Confocal microscopy and FRET measurements

A Zeiss LSM 510 Meta laser scanning microscope equipped with a Plan-Apochromate 63x/1.4 oil immersion objective was used to examine images of 1 μm thick slices of live cycling HeLa cells grown in glass bottom culture dishes (MatTek Inc., USA). Fluorescence energy transfer (FRET) was determined by modifying the general equations given by Matyus (1992) as described in Baynton *et al *(2003) [42,47]. FRET occurs if I_2 _– I_1_(I_D2_/I_D1_) – I_3_(I_A2_/I_A3_) >0 where I represents intensities in three channels given in arbitrary units between 0 and 250. Normalised FRET is defined as *N*_FRET _= FRET/(I_1 _× I_3_)^1/2 ^[48]. Intensities were measured as follows: channel 1: I_1, A1, D1 _= excitation (ex.) at λ = 458, detection (det.) at 470 nm <λ>500 nm (ECFP); channel 2: I_2, D2, A2 _= ex. at λ = 458 nm, det. at λ>560 nm; channel 3: I_3, D3, A3_= ex. at λ = 514 nm, det. at λ >560 nm (EYFP). I_D1, D2, D3 _and I_A1, A2, A3 _are determined separately for cells transfected with only ECFP- and EYFP-fusion proteins respectively, under the same settings and at the same levels of fluorescence intensities (I_1 _and I_3_) as co-transfected cells.

### Clonogenic assays

6 well plates were seeded with 2 – 5 × 10^4 ^cells per well and incubated overnight prior to transfecting with 6 μg per well of plasmid DNA. The medium was replaced with fresh 24 hours post-transfection and G418 added to a final concentration of 1.0 mg/ml at 48 hours post-transfection. Medium containing G418 was replaced as necessary until 10 to 14 days post-transfection. Cells were then washed twice in PBS, fixed for 15 minutes in ice-cold methanol, then stained with 10% (w/v) Giemsa for 15 minutes and rinsed in water.

## Authors' contributions

EW conceived of the study, drafted the manuscript, carried out the two-hybrid analysis and the clonogenic assays. EW and JRGP made the expression constructs and performed the immunofluorescence analysis. JRGP performed the immunoprecipitation experiments. MO carried out the FRET experiments and analysis. All authors helped to draft and approved the manuscript.
